# First *Cryptosporidium* outbreak in Hungary, linked to a treated recreational water venue in 2015

**DOI:** 10.1017/S0950268818003138

**Published:** 2018-12-03

**Authors:** J. Plutzer, K. Kelen, E. Varga, I. Kucsera, G. Reusz, A. J. Szabó, Á. Fehér, R. M. Chalmers

**Affiliations:** 1 Department of Water Hygiene, National Public Health Institute, Budapest, Hungary; 2 1st Department of Paediatrics, Semmelweis University, Budapest, Hungary; 3 Public Health Department, Government Office of Somogy county, Kaposvár, Hungary; 4 Department of Parasitology, National Public Health Institute, Budapest, Hungary; 5 Hungarian Academy of Sciences and Semmelweis University Pediatric and Nephrology Research Lab, Budapest, Hungary; 6 Department of Public Health, Semmelweis University, Budapest, Hungary; 7 Cryptosporidium Reference Unit, Public Health Wales Microbiology and Health Protection, Singleton Hospital, Swansea, UK

**Keywords:** Children's swimming pool, *Cryptosporidium*, jetted whirlpool, outbreak

## Abstract

In June 2015, an outbreak of cryptosporidiosis with 35 cases (23 probable and 12 laboratory-confirmed) occurred among 191 attendees of a residential rehabilitation holiday for paediatric organ transplant patients (*n* = 49) and their families at a hotel in Somogy county, Hungary. The overall attack rate was 18%. Most of the cases were transplanted children who experienced severe acute disease and required adjustment to their tacrolimus immunosuppression. A retrospective case-control study suggested an association between recreational water exposures and illness: cases were seven times more likely than controls to have swum in the children's pool (odds ratio 7.17; 95% confidence interval 2.9–17.2; *P* < 0.0001) and five times more likely to have used the jetted whirlpool (odds ratio 5.25; 95% confidence interval 2.1–13.1; *P* < 0.0001). This was the first outbreak of cryptosporidiosis in Hungary and it is especially unfortunate that it affected vulnerable children who experienced severe symptoms. *Cryptosporidium* presents specific infection control difficulties in treated recreational water venues; the link to a whirlpool is unusual and highlights the importance of the age-appropriate use of these facilities and reminding users not to immerse their heads or swallow the water. Cryptosporidiosis is more commonly linked to children’ pools where improved bather hygiene and promoting exclusion of diarrhoea cases could help to avoid similar outbreaks.

## Introduction

Infection with the protozoan parasite *Cryptosporidium* causes the gastrointestinal disease cryptosporidiosis which is characterised by watery diarrhoea, abdominal pain, nausea and/or vomiting and low-grade fever [1]. Symptoms can range from mild to severe and usually last for up to 2–3 weeks and can recur [2]. Oocysts may be shed for 2 weeks after symptoms have ceased, with long-term asymptomatic carriage also reported [3]. Illness is usually self-limiting, but in patients with certain T-cell related immuno-deficiencies, it can be severe and sometimes life-threatening [4]. All ages can be affected, but more cases are reported among children, especially the under 5-years-old, than adults [5].

Oocysts, which are 4–6 µm in diameter, are shed in stools in high numbers (10^6^ to 10^7^ per gram of stool) during acute illness and can infect immediately new hosts directly or via fecally-contaminated water and food [5, 6]. The infectious dose is as low as 10 oocysts [5]. *Cryptosporidium* oocysts can survive for months in water or moist soil and resist harsh environmental conditions [5].

*Cryptosporidium* continues to be the dominant aetiology of recreational water-associated outbreaks of gastrointestinal illness. The majority of all treated recreational water venue-associated outbreaks reported were caused by *Cryptosporidium* in the USA and UK [7, 8], because of the extreme chlorine-tolerance of this parasite; it can survive chlorination of drinking water and swimming pools. Furthermore, swimming pool filters may not be designed to remove oocysts, which can break through, although removal efficiency can be improved with coagulation [7, 8].

The number of reported *Cryptosporidium* infections varies greatly between countries in Europe and only a few cases are reported from Eastern Europe; the causes of variation may include true differences in risk exposures and susceptibility, variable provision and access to health care systems, case definition, laboratory diagnosis and reporting of cases [9]. The quality of the drinking water and sewage treatment systems, use and management of recreational waters and the nature of investigations impacts the assessment of the waterborne disease burden and identification of outbreaks [10]. Although *Cryptosporidium* is notifiable in Hungary, a mean of 14 cases was reported annually from 2010 to 2014 [11].

On 9 July 2015, a diarrhoea patient, who was among a group visiting a hotel from 14 to 20 June 2015, submitted a complaint to the local health department of Government Office of Somogy county, that many other diarrhoeic cases occurred among hotel guests. An investigation was initiated by the local Public Health Authority. We describe the first investigation of a cryptosporidiosis outbreak in Hungary, which occurred among vulnerable children and was linked to a treated recreational water venue, serving to alert others who lack awareness of the risk posed by *Cryptopsoridium*.

## Methods

Table 1 provides a timeline of the investigation.

**Table 1.** Timeline of the hotel inspections and outbreak investigation in relation to a holiday for transplanted patients and their families, 14–20 June 2015

Date	Routine inspections	Outbreak investigation
16 June		Onset of symptoms of the first cases and presentation to the holiday doctor or to the local physician
21–23 June		Onset of symptoms of most cases
29 June	Hotel pools tested for chemical and bacteriological parameters (satisfactory)	
2 July	Hotel swimming pool inspected (satisfactory)	Onset of symptoms of the last case
9 July		Complaint to local health department about a diarrhoea outbreak at the hotel; formation of the outbreak control team; start of interviewing cases for descriptive epidemiology
14 July		Swimming pool and hotel kitchen inspection; provision of advice to prevent spread in the hotel
15 July		Start of interviewing hotel residents for analytical epidemiology
17 August		*Cryptosporidium* investigations of pool water

### Case finding

It rapidly emerged during a discussion with the hotel that diarrhoea had been reported among attendees of a residential rehabilitation holiday for paediatric organ transplant patients, their families and volunteer helpers. These comprised 191 persons of whom 49 were transplanted children. The camp was organised by a Transplant Foundation which was able to provide a full list of participants and their contact details enabling interview either by phone, e-mail or face-to-face. Information was gathered whether they developed symptoms, date of onset, age, sex, clinical history and treatment. Patients were asked to submit stool samples.

### Descriptive epidemiology

Microbiological and environmental findings were summarised descriptively and cases described by a person, time and place (date of onset of symptoms, pool use, age and sex distribution, symptoms and treatment) using the following case definition:

*Probable case*: accommodated in the hotel on 14–20 June 2015 and had diarrhoea and/or gastrointestinal symptoms within 3 weeks of attendance.

*Confirmed case*: as probable with reference laboratory confirmation of *Cryptosporidium* in the stool samples.

### Analytical epidemiology

The outbreak investigation (15 July 2015) aimed to understand the risk factors for illness in this group. Additional information was collected through an interactive questionnaire administered either by phone, e-mail or face-to-face including data on the presence of symptoms and potential exposures (water supply, food provided by the hotel and recreational activities such as swimming). This short questionnaire was intended for all hotel guests during 15–20 July 2015, whether or not they had clinical symptoms and the data used for a retrospective, unmatched, case-control study to determine the exposures associated with illness. Cases were individuals who attended the rehabilitation holiday on 14–20 June 2015 and subsequently developed diarrhoea and/or vomiting and/or abdominal cramps within 3 weeks of attendance. Controls were individuals who attended the rehabilitation holiday on 14–20 June 2015 and subsequently did not develop diarrhoea and/or vomiting and/or abdominal cramps within 3 weeks of attendance.

### Statistical methods

Two by two tables were used to evaluate the association between exposure and gastrointestinal symptoms. Odds ratios were calculated using the OpenEpi v.2.3.1.program [12].

### Stool microbiology

The 35 symptomatic patients who submitted stool samples for testing did so within 1 week of onset of symptoms and were tested first for rotavirus, adenovirus and cytomegalovirus and then for bacteria (*Salmonella*, *Shigella, Campylobacter, Yersinia, Escherichia coli, Clostridium difficile*) using accredited standards of the National Public Health Institute. Following negative findings, stools were tested for *Cryptosporidium* using an immunochromatographic lateral flow (ICLF) test (Rida Quick *Cryptosporidium*, R-Biopharm, Germany) and positive results were corroborated later microscopically by Kinyoun staining at the national reference laboratory. Once *Cryptosporidium* was identified as the aetiological agent, as a precaution from 15 July 2015, all transplanted and other children who attended the rehabilitation holiday were tested for *Cryptosporidium* (all together 52 persons).

### Environmental investigations

The hotel's swimming pool complex comprised a swimming pool (23–27 °C, ⩾1.2 m depth), a children's pool (28–32 °C, ⩽0.6 m depth) and a jetted whirlpool (1450 l, 28–32 °C). The hotel had its own kitchen serving a buffet breakfast and daily menu for lunch and dinner. The hotel had been inspected routinely by local authorities on 2 July and subsequently on 14 July 2015 (after the recognition of the outbreak). Information was obtained about facilities, menus and about the mode of food processing, storage and kitchen hygiene as well as observations to determine compliance with regulations and standards [13–15], review test logs, response procedures and testing water quality parameters and communication of inspection results.

Routine collection of pool water samples for complex physico-chemical (temperature, turbidity, pH, conductivity, ammonium, chemical oxygen demand, chloride, free residual chlorine, combined chlorine, total alkalinity, total hardness) and bacteriological (aerobic colony count at 37 °C 24 h, Total faecal coliform bacteria/*Escherichia coli, Pseudomonas aeruginosa, Legionella, Micrococcus, Enterococcus, Staphylococcus aureus*) investigations had been taken on 29 June 2015. Samples were tested using relevant Hungarian regulations and standards [13–15].

Water samples were taken from the swimming pools of the hotel for parasitological (*Giardia, Cryptosporidium*) investigations on 17 August 2015 (1 month after the recognition of the outbreak). *Cryptosporidium* tests on the pool water were performed according to US Environmental Protection Agency Method 1623: *Cryptosporidium* and *Giardia* in Water by Filtration/IMS/FA. Pool waters were sampled by 2 µm nominal pore size microfibre filtration [16] until the filters clogged, achieving sample volumes of 35 l of children's pool water and 22 l of whirlpool water.

## Results

### Descriptive analysis

**Fig. 1.**
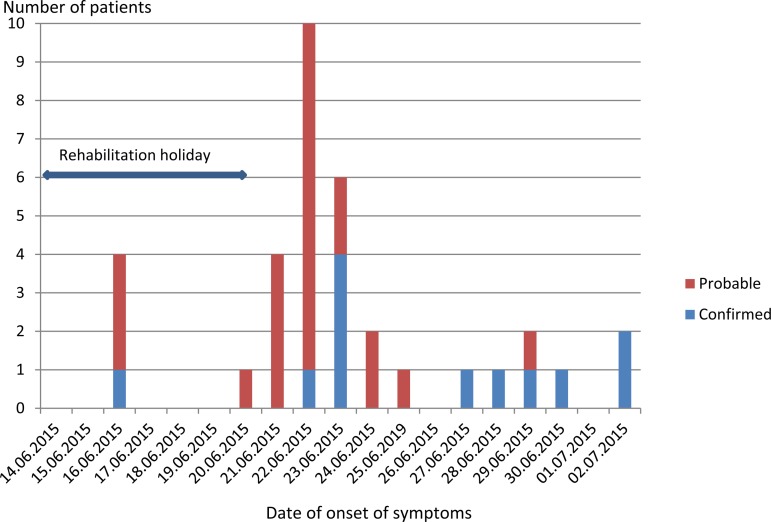
Epidemic curve of a gastroenteritis outbreak invovling *Cryptosporidium* involving vulnerable children in Somogy county, Hungary, 2015.

The investigation identified 35 cases: 12 confirmed and 23 probable. The overall attack rate was 35/191 (18.3%) of attendees at the rehabilitation holiday. Of the 191 attendees, 49 were transplanted children, 89 siblings and parents and 53 volunteer helpers. Fourteen (40%) cases were male, 21 (60%) female. Cases ranged in age from 9 months to 59 years; 19 (54%) of the cases were children aged 0–10 years (all of them transplanted), 10 (29%) aged 10–15 years and 6 (17%) were adults aged 38–59 years. Dates of onset of illness ranged from 16 June to 2 July (Fig. 1). Four people (two children and two adults in one family) developed diarrhoea just 2 days after arrival at the hotel, one of them (a transplanted child) was a confirmed case who probably acquired infection before arrival. One transplanted patient initially appeared to have developed symptoms more than 12 days after leaving the hotel (7 July 2015), but on further questioning their diarrhoea began initially 2 weeks earlier (23 June 2015), but she visited the doctor later when symptoms reappeared.

Symptoms included prolonged diarrhea, nausea and vomiting, which in many cases led to dehydration. Those affected typically had a large volume, watery stools. Both immunosuppressed and healthy children were affected, however, the symptoms were less severe amongst otherwise healthy children. The medical care of the majority of the patients was carried out at a single centre. Eleven out of the 12 *Cryptosporidium* confirmed stool samples were from transplanted children 1–10 years of age, including six kidney, three liver, one lung and one liver and kidney transplantation. No other pathogens were found. All of these children received tacrolimus immunosuppression and they averaged 3.81 (±1.8) years post-transplantation. Of the 11 transplanted children with *Cryptosporidium*, seven were admitted to hospital for an average of 8.85 (±3.26) days and six received parenteral rehydration with electrolytes. Graft function remained within the normal range in each case. However, increased trough blood level of tacrolimus was noticed in six children warranting a dose reduction in five cases. Eight children were treated with nitazoxanide (200 mg/12 h; 3 days). Children in both the treated and untreated groups became asymptomatic and we could not find a difference in the time of the recovery of nitazoxanide treated and untreated children. Chronic infection could not be detected a year later in any of the children.

The holiday was organised by the Transplant Foundation in order to promote a successful return after a chronic disease into the community life. Sports programs had a key role during the holiday, where swimming, tennis and table tennis were taught with the supervision of trainers. Because of the immunosuppressive therapy, a doctor was continuously present to pay attention to transplanted children and infection control. The hotel was suitable for accommodating around 200 people and besides the participants of the camp, there were 22 other guests that time in the hotel. These 22 guests had training for a swimming competition and they used the same swimming pool. However, they used solely the main pool in the afternoons when the rehabilitation holiday participants were not allowed to enter the swimming pool. The competitors are regular users of the hotel swimming pool and their interview was performed besides the participants of the camp either by phone, e-mail or face-to-face when the analytical epidemiology started (15 July 2015). None of these competitors had gastrointestinal symptoms.

### Analytical epidemiology

A total of 138/191 (72.3%) people participated in the retrospective case-control study by answering the questionnaire. There were 34 cases and 104 controls. This revealed that cases were more likely than controls to have swum in the children's pool (odds ratio 7.17; 95% confidence interval 2.9–17.2) and cases were more likely than controls to have used the whirlpool (odds ratio 5.25; 95% confidence interval 2.1–13.1) (Table 2). There was no association with using the main hotel swimming pool (odds ratio 1.36; 95% confidence interval 0.6–3.0) or swimming in Lake Balaton (odds ratio 1.13; 95% confidence interval 0.5–2.4) (Table 2). The role of the food was discarded as all hotel residents had eaten the same food.

**Table 2.** Exposure data for case-control study participants

Exposure	Cases *n* = 34		Controls *n* = 104		Odds ratio	95% CI
	Number	%	Number	%		
Children's pool	25	73.5	29	27.9	7.17	2.9–17.2
Whirlpool	27	79.4	44	42.3	5.25	2.1–13.1
Swimming pool	23	67.6	63	60.6	1.36	0.6–3.0
Swimming in Lake Balaton	18	52.9	52	50.0	1.13	0.5–2.4

### Environmental investigations

On-site inspection by local government staff on 14 July 2015 of the swimming pools and whirlpool revealed that the water recirculation system worked properly, irregularities were not found and operations were according to the relevant regulations. A complete change of waters in all pools had been performed on 14 May 2015 at the beginning of the season. Pool water replacement (30 l/bather/day) was the part of the water treatment regime to limit the accumulation of pollutants from bathers, disinfection by-products and other dissolved chemicals. The children's pool had an ASTRAL PTK 800 mm filter with 1 m filtration bed, 0.5 m^2^ surface area, grain sizes 0.4–0.8 mm sand (617 Kgs) and 1–2 mm gravel (109 Kg's), providing a medium flow rate of 18–22 m^3^/h. The whirlpool had a Krispol SSB 900 mm sand filter with 1 m filtration bed, 0.45 m^2^ surface area and medium flow rate 18–22 m^3^/h. Backwashing of the filters was undertaken a minimum of twice a week and a complete change of the water in the whirlpool was performed every other day. Coagulant dosing was achieved by partly automated fine dosing of polyaluminium chloride (Dinax Flock F). The disinfectant was sodium hypochlorite (Dinax Klorin F). Dosing of disinfectant and pH control chemicals (Dinax Mínusz F) were fully automated and free residual chlorine targeted and achieved 0.3–0.5 mg/l and pH targeted and achieved 7.0–7.2. Self-control chemical tests (residual disinfectant and pH levels) and bacteriological and chemical tests were all negative or within the acceptable range. *Cryptosporidium* and *Giardia* tests on the pool water were all negative.

The kitchen inspection revealed no evidence of poor hygiene or poor temperature control during the preparation of food.

On 14 July 2015, the local government staff provided recommendations on disinfection procedures to the hotel to prevent spread, which include backwashing and disinfection (hyperchlorination) of the filters at the whirlpool and children's pool, disinfection and deep cleaning of the walls of the pools and surrounds and a complete change of the water. These control measures were implemented immediately.

## Discussion

This outbreak of 35 cases of gastrointestinal disease, occurring within 3 weeks of the transplant rehabilitation holiday, solely among that group of residents at the hotel and mainly among children, strongly suggested a common source and a waterborne outbreak. The clinical symptoms and incubation period were typical of *Cryptosporidium* infection and adversely affected vulnerable children, some of whom experienced upset in the metabolism of tacrolimus indicated by raised trough blood levels and the dosage had to be decreased. Similar effects have been reported following other gastrointestinal infections [17–19]. Immunosuppressed patients are at higher risk for more severe and more prolonged cryptosporidiosis and in this outbreak, seven children were admitted to hospital where six received parenteral rehydration with electrolytes. Even through illness did not last more than 3 weeks, transplanted children may carry the infection and symptoms reappear, therefore, continuous control measures are necessary [17]. Diarrhea is a common problem amongst organ transplant patients and may lead to dehydration, increased toxicity of medications and graft rejection. In case of persistent diarrhoea, besides common pathogens in this patient group (*Clostridium difficile,* CMV, Norovirus) *Cryptosporidium* should also be suspected and tested [17, 18].

One of the main challenges in this investigation was the timing of *Cryptosporidium* sampling from swimming pools, as the pools had been disinfected and water changed before this could be done. The absence of *Cryptosporidium* in the samples cannot be considered fully representative of the status of the water environment during the holiday and the sampling from pools gave, therefore, no indication of the source of transmission for the contamination. Timely sampling would require co-ordination across multiple organisations; testing water samples for *Cryptosporidium* is expensive and by the time sampling can be arranged may not be relevant anyway [7]. Therefore the epidemiological study was very useful to pinpoint the cause of the outbreak as waterborne and demonstrated a significant association between gastroenteritis and using the children's pool and whirlpool. While according to the literature children's pools are commonly associated with *Cryptosporidium* outbreaks, because they are most likely to become faecally contaminated and the treatment may be inefficient at removing oocysts, it is unusual for whirlpools to be linked to cases. The main risk behaviours by children in swimming pools include swallowing pool water (often indicated by frequent head immersion) [7], but whirlpools are usually used by adults who do not normally immerse their heads and swallow the water in this type of pool.

A large proportion of outbreaks of *Cryptosporidium* infection are probably never recognised or reported [20]. This outbreak occurred among a specific group of residents at a hotel with an assigned doctor, facilitating case finding and collection of information and contributed to the recognition of the outbreak which ranks the first outbreak of *Cryptosporidium* infection ever reported in Hungary. The strengths of this outbreak investigation are the high rate (72.3%) of completion of the analytical epidemiology questionnaires and the prompt collection of stool samples, which provided a rapid microbiological diagnosis and fast response of the doctors of transplanted patients. The use of the ICLF assay facilitated rapid testing for *Cryptosporidium* in a setting where this parasite is rarely looked for. Although positive reactions were confirmed microscopically, this test has been shown to be less sensitive than enzyme immunoassay and some positive cases may have been undiagnosed [21]. Ideally, surveillance and outbreak investigations should be supported by genotyping to identify *Cryptosporidium* species and subtypes, enabling epidemiological predictions of transmission. However, such investigations are not undertaken routinely in Hungary, therefore, stool samples were not kept for genetic analysis. In this case-control study, epidemiologists collected data in both the exposed and unexposed groups from past records and did not follow up with patients over a long period of time. All the events such as exposure, latent period and the development of gastrointestinal disease were retrospective and therefore information bias including exposure or outcome assessment could not be controlled. This can negatively impact the accuracy of this type of study.

### Interpretation

*Cryptosporidium* presents specific infection control difficulties in treated recreational water venues because it is resistant to normal chlorine levels used for pool disinfection against bacteria. Removal of oocysts from pool water relies on good circulation with optimal filtration and flocculation which demand extra attention. The treatment of the children's pool and whirlpool may have been compromised by lower than optimal pH for chlorinated pools (pH 7.2–7.4) for the coagulant to be most effective [22]. Bathers are at risk if they ingest pool water that has been faecally contaminated by someone infected with *Cryptosporidium.* The children's pool and the whirlpool provided a higher risk of infection since small pools provide less dilution of contaminants and user behaviour such as head immersion and swallowing pool water increases the risk of exposure.

Outbreak reporting and descriptions are important to understand the epidemiology of *Cryptosporidium* and for increasing awareness, prevention of future outbreaks and the development of targeted interventions such improved design and operation of pool water treatment. It is unusual to have cases of cryptosporidiosis linked to a whirlpool and this outbreak re-enforces the importance of appropriate use of these facilities by age-restricting their use and reminding users not to immerse their heads or swallow the water. Cryptosporidiosis is more commonly linked to children's pools. Bather hygiene needs to be re-enforced, reminding swimmers to use toilet facilities and to shower before swimming and about the ‘no swim rule’ if they have suffered from vomiting or diarrhoea within 2 weeks. Furthermore, supplementary verbal reinforcement in relation to faecal contamination could be appropriate. Vulnerable children should be advised not to put their heads under the water in swimming pools and avoid the use of whirlpools. The management of infectious diseases among children who undergo transplantation requires a continuum of care. For specific groups where vulnerable people are using shared facilities, health checks should be made first.
